# Preeclampsia: Recent Advances in Predicting, Preventing, and Managing the Maternal and Fetal Life-Threatening Condition

**DOI:** 10.3390/ijerph20042994

**Published:** 2023-02-08

**Authors:** Kai-Jung Chang, Kok-Min Seow, Kuo-Hu Chen

**Affiliations:** 1 Department of Obstetrics and Gynecology, Taipei Tzu-Chi Hospital, The Buddhist Tzu-Chi Medical Foundation, Taipei 231, Taiwan; tch36289@tzuchi.com.tw; 2 Department of Obstetrics and Gynecology, Shin Kong Wu Ho-Su Memorial Hospital, Taipei 111, Taiwan; m002249@ms.skh.org.tw; 3 Department of Obstetrics and Gynecology, National Yang-Ming Chiao-Tung University, Taipei 112, Taiwan; 4 School of Medicine, Tzu-Chi University, Hualien 970, Taiwan

**Keywords:** preeclampsia, gestational hypertension, pregnancy induced hypertension, proteinuria

## Abstract

Preeclampsia accounts for one of the most common documented gestational complications, with a prevalence of approximately 2 to 15% of all pregnancies. Defined as gestational hypertension after 20 weeks of pregnancy and coexisting proteinuria or generalized edema, and certain forms of organ damage, it is life-threatening for both the mother and the fetus, in terms of increasing the rate of mortality and morbidity. Preeclamptic pregnancies are strongly associated with significantly higher medical costs. The maternal costs are related to the extra utility of the healthcare system, more resources used during hospitalization, and likely more surgical spending due to an elevated rate of cesarean deliveries. The infant costs also contribute to a large percentage of the expenses as the babies are prone to preterm deliveries and relevant or causative adverse events. Preeclampsia imposes a considerable financial burden on our societies. It is important for healthcare providers and policy-makers to recognize this phenomenon and allocate enough economic budgets and medical and social resources accordingly. The true cellular and molecular mechanisms underlying preeclampsia remain largely unexplained, which is assumed to be a two-stage process of impaired uteroplacental perfusion with or without prior defective trophoblast invasion (stage 1), followed by general endothelial dysfunction and vascular inflammation that lead to systemic organ damages (stage 2). Risk factors for preeclampsia including race, advanced maternal age, obesity, nulliparity, multi-fetal pregnancy, and co-existing medical disorders, can serve as warnings or markers that call for enhanced surveillance of maternal and fetal well-being. Doppler ultrasonography and biomarkers including the mean arterial pressure (MAP), uterine artery pulsatility index (UtA-PI), and serum pregnancy-associated plasma protein A (PAPP-A) can be used for the prediction of preeclampsia. For women perceived as high-risk individuals for developing preeclampsia, the administration of low-dose aspirin on a daily basis since early pregnancy has proven to be the most effective way to prevent preeclampsia. For preeclamptic females, relevant information, counseling, and suggestions should be provided to facilitate timely intervention or specialty referral. In pregnancies complicated with preeclampsia, closer monitoring and antepartum surveillance including the Doppler ultrasound blood flow study, biophysical profile, non-stress test, and oxytocin challenge test can be arranged. If the results are unfavorable, early intervention and aggressive therapy should be considered. Affected females should have access to higher levels of obstetric units and neonatal institutes. Before, during, and after delivery, monitoring and preparation should be intensified for affected gravidas to avoid serious complications of preeclampsia. In severe cases, delivery of the fetus and the placenta is the ultimate solution to treat preeclampsia. The current review is a summary of recent advances regarding the knowledge of preeclampsia. However, the detailed etiology, pathophysiology, and effect of preeclampsia seem complicated, and further research to address the primary etiology and pathophysiology underlying the clinical manifestations and outcomes is warranted.

## 1. Introduction

**Figure 1:**
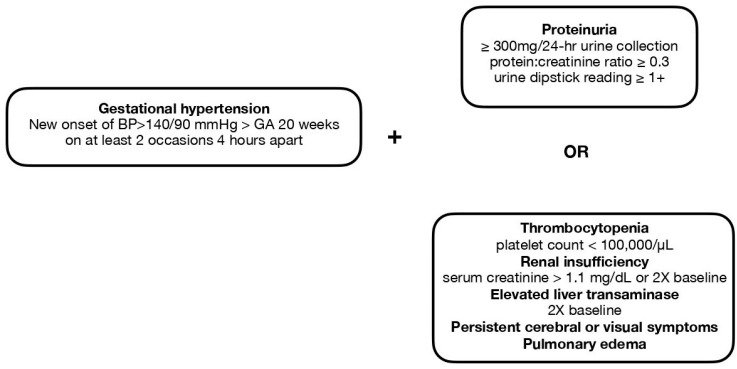
The definitions of gestational hypertension (pregnancy-induced hypertension) and preeclampsia.

Hypertensive disorder during pregnancy poses a substantial threat to both maternal and fetal health conditions [1]. Preeclampsia is one of the most well-known medical conditions that belong to this disease spectrum, which also accounts for one of the most common documented gestational complications, with a prevalence of approximately 2 to 15% of all pregnancies [2,3]. It is depicted as a gestational condition with a hypertensive disorder diagnosed after 20 weeks of gestation and coexisting proteinuria or generalized edema, and certain forms of hematologic disorders such as thrombocytopenia or signs of end organ damage including renal impairment, abnormal liver function, pulmonary edema, and cerebral and visual disturbance [4,5]. The definitions of gestational hypertension (pregnancy-induced hypertension) and preeclampsia are shown in Figure 1. Serious or long-term complications may result when preeclampsia turns into a severe type or is left without being sufficiently treated. Multiorgan involvement may be seen in such cases, and the impairment of uteroplacental perfusion could potentially lead to gestational complications and poor fetal outcomes including intrauterine fetal growth restriction and preterm delivery. As the situation worsens, it may become life-threatening for both the mother and the fetus, in terms of increasing the rate of mortality and morbidity [5].

One straightforward way to categorize preeclampsia is to subdivide it into early-onset and late-onset groups in accordance with the gestational age (GA). The cutoff point is usually set as GA 34 weeks or GA 37 weeks, and we can subcategorize preeclampsia into the early-onset (GA < 34 weeks), late-onset (GA ≥ 34 weeks), preterm (GA < 37 weeks), and term (GA ≥ 37weeks) subgroups (Table 1). The diagnoses made at different timings during the pregnancy course may suggest different pathophysiologic and etiologic pathways [6].

Preeclampsia should be viewed as a disease spectrum in which different subtypes may vary greatly in disease mechanisms and clinical presentations. The status of a previously normotensive pregnant woman developing new onset of hypertension after GA 20 weeks is termed “gestational hypertension” or “pregnancy induced hypertension”. If aside from gestational hypertension, a patient is also noted with proteinuria, thrombocytopenia, impairment in renal or liver function, cerebral symptoms, visual symptoms, or pulmonary edema, then she meets the diagnostic criteria of preeclampsia (Figure 1). In terms of severity, preeclampsia could be classified as “nonsevere” or “severe” types (Table 2), with the latter group exhibiting clinical features including blood pressure exceeding 160/100 mmHg, headache, visual disturbances, upper abdominal pain, oliguria, elevated serum creatinine, thrombocytopenia (<100,000/µL), elevated level of liver enzymes, fetal growth restriction, pulmonary edema, onset at an early gestational age, and the presence of convulsion (eclampsia) [7].

Although the definite cause of preeclampsia remains unknown to date, several hypotheses have been made to explain its pathophysiology. One of the most commonly accepted theories is the two-stage model, which proposes that inadequate trophoblast invasion would lead to shallow placentation and subsequent poor uteroplacental perfusion (stage I), thus causing widespread endothelial dysfunction and systemic clinical manifestations (stage II) [8]. The window between the first and second stages provides an optimal opportunity for prediction during the subclinical phase [5]. Known as a safe and effective drug in the prevention of pregnancy-related vascular disorders including but not limited to preeclampsia, aspirin has been applied for preeclampsia prevention with a low dosage starting as early as before GA 16 weeks and until approximately GA 36 weeks [9]. Nevertheless, non-pregnant women throughout the world enjoy the privilege of early prevention and intervention. Even if they do, sometimes preeclampsia may still develop. The only definite solution for preeclampsia is the delivery or termination of pregnancy. When a diagnosis is made, antihypertensive medication is, however, one of the most important treatments before delivery. Fluid control, prevention, and treatment for end organ damage should be applied as well [10].

Due to the notable prevalence and influence of preeclampsia in pregnancy, an understanding of preeclampsia, as thorough as possible, is crucial. The review aimed to summarize existing studies in the literature to explore the epidemiology, etiology (risk factors), socioeconomic burdens, pathophysiologic mechanisms, prediction, prevention, and treatment of preeclampsia. The cutting-edge studies will be analyzed and integrated into this review to provide state-of-the-art knowledge.

## 2. Materials and Methods

### Searching Terms and Strategies in the Literature

**Figure 2:**
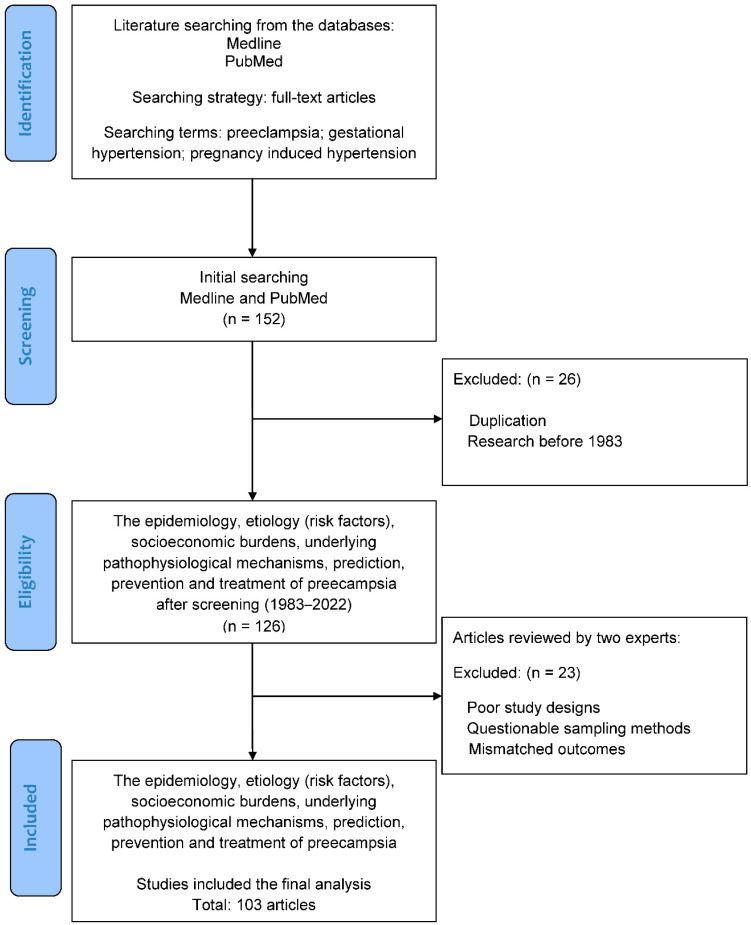
Flowchart of database searching, screening, and inclusion of the references selected from the literature.

The literature was searched to identify basic and clinical studies, which investigated the epidemiology, etiology (risk factors), socioeconomic burdens, and underlying pathophysiological mechanisms of preeclampsia, along with its prediction, prevention, and treatment. Figure 2 illustrates the flowchart of database searching, screening, and inclusion of the references that we selected from the literature. In this review, all of the articles were retrieved from the databases Medline and PubMed using the search terms “preeclampsia”, “gestational hypertension”, and “pregnancy induced hypertension” for the research topic. For screening and selection in the next stage, only full-text articles were considered for inclusion in further analysis. In the second stage, the articles published before 1983 were excluded to ensure the novelty of the current review. Duplicated articles were also excluded. From a total of 152 articles identified in the screening process, 126 potential articles (1983–2022) met the criteria for inclusion.

Hereafter, two experts in the field independently inspected the contents of articles including demographics, research designs, and outcomes, and identified eligible basic and clinical studies for inclusion. The solicited articles with poor research designs, questionable sampling methods, or mismatched outcomes would be excluded at this stage. The discrepancies between the experts were discussed via mutual communication to reach a consensus. All eligible studies were included in the review using the search terms and strategies (identification from the database, screening of the studies, selection of potential articles, and final inclusion). Finally, a total of 103 articles were collected for review from 152 articles identified in the initial search.

## 3. Epidemiology, Etiology (Risk Factors), and Economic Burden of Preeclampsia

### 3.1. Epidemiology and Risk Factors

Preeclampsia is a gestational disorder affecting women worldwide from different nations, ethnicities, age groups, etc. Overall, a prevalence rate of approximately 2 to 15% of pregnant women is documented, with an average prevalence rate of approximately 4.6% [2,3,11]. The pathophysiology of preeclampsia is complex and remains incompletely unveiled, which makes it sensible that its prevalence and traits would vary under different circumstances. In other words, different populations with preeclampsia may display different prevalence, patterns, or distribution of risk factors and pregnancy outcomes. The study of its epidemiology and risk factors could thereby demonstrate its complexity and heterogeneity. Table 3 presents a list of risk factors for preeclampsia.

### 3.2. Race and Ethnicity

In a cross-sectional study conducted by Yang et al., a thorough comparison between the characteristics of preeclampsia among the Chinese and Swedish populations was made. The study included a total of 634,689 pregnancies, among which the Chinese and the Swedish exhibited similar prevalence rates of approximately 2 to 3%. However, there were marked variabilities in the other descriptive results. The maternal age, mean body mass index (BMI), and obesity rates were higher in Sweden, while more nulliparous women and cesarean deliveries were identified in their Chinese counterparts. The disease extent and pregnancy outcomes also differed. Mild preeclampsia was more common in the Swedish population, while there were more severe cases in China. The Chinese also had overall higher rates of stillbirth, preterm birth, and low birth weight. Ethnicity, lifestyle, metabolic perturbations, genetic factors, and seeking medical help may all contribute to these variabilities [11]. While race is a potential contributory factor, it may not be completely persuasive in this scenario since the Swedish comprised relatively richer ethnicities whereas the Chinese were primarily Hans.

The role of race in preeclampsia has been investigated in various studies. A review article written by Zhang et al. pointed out that African Americans had a higher rate and severity of preeclampsia, which was likely related to, if not directly resulting from, multifactorial causes including previous history of preeclampsia, system lupus erythematosus, sickle cell anemia, gestational diabetes mellitus, and a history of chronic hypertension [12]. Another study conducted by Ghosh et al. suggested that non-Hispanic women had higher odds of developing preeclampsia and had greater severity of disease, compared with Hispanic women and Asian/Pacific Islanders. An expert review written by Johnson also mentioned a higher risk of preeclampsia among Black, Native American, and Native Alaskan races. Nevertheless, it is worth noting that research focusing on the role of race or ethnicity in the disparities of preeclampsia shares some common limitations. Firstly, race or ethnicity is not a scientifically biological or genetic trait; rather, it is more often self-reported and thus may become subjective. Secondly, a person could belong to more than one racial or ethnic group instead of being assigned to one single category. Thirdly, a standardized method of classification used in medical research may fail to reflect the cultural, lingual, or historical origins and distributions in reality. Instead of serving as a direct or independent factor, the role that race or ethnicity plays in preeclampsia may correspond to the reflection or marker for the influence of cultural, socioeconomic, or healthcare resources, etc. [13].

### 3.3. Age

Many real-world statistics suggest that advanced or extremely young maternal age is an important risk factor for preeclampsia. Furthermore, these mothers at risk may also face more adverse maternal and neonatal outcomes and hence should raise special concerns throughout their pregnancy courses.

A cohort study that included preeclamptic individuals from 1998–2014 in the U.S. suggested that women at extreme ages (<25 years or >45 years) tended to develop severe morbidities. Women younger than 25 years of age had a significantly higher rate of developing eclampsia. On the other hand, women more than 45 years old were more likely to suffer from acute heart failure and acute kidney injury (acute renal failure). The results suggested that both extremely young and elderly mothers were exposed to a greater threat during gestation but possibly from different perspectives [14].

Some studies focused mainly on the advanced maternal age (AMA) groups. A registry-based study in Finland suggested that women older than 35 years exhibited more preeclampsia, early and late preterm deliveries, cesarean deliveries, and poorer neonatal outcomes [15]. In a retrospective cross-sectional study in Indonesia conducted in 2016-2017, preeclamptic women over 35 years of age developed more severe complications in general, with postpartum hemorrhage in particular, while no significant increase in the developments of HELLP syndrome, visual disturbances, pulmonary edema, or eclampsia was identified. Regarding neonatal outcomes, there were more preterm deliveries (GA < 37 weeks), intrauterine growth restrictions, neonatal asphyxia, and neonatal infections in the group of advanced-age women [16].

### 3.4. Parity

Nulliparity has long been classified as a risk factor, as it may triple the risk for preeclampsia [17]. Some studies have concluded that nulliparous women were found with a higher percentage of preeclampsia compared to other cohorts [18]. Many hypotheses attribute this to immunological reasons. A feasible explanation is that a suboptimal maternal adaptation to fetal or paternal alloantigens may indirectly result in impaired uteroplacental perfusion, which accounts for the pathogenesis of preeclampsia [19]. Nulliparous women were also proposed to endure an “angiogenic imbalance”, manifested as a higher circulating sFlt1 level and sFlt1/PIGF ratio, which may also contribute to their tendency of developing preeclampsia [20]. From a fundamental viewpoint, meanwhile, there may be little difference between nulliparous and multiparous women regarding other risk factors including AMA, diabetes mellitus, multifetal gestations, etc. [21].

### 3.5. Obesity

Obesity is an alarming issue in the modern world and has proven to confer many hazards to human health. With time, it has raised concerns in both developed and developing countries. As one of the leading attributable risk factors, the mechanism of how obesity could potentially lead to preeclampsia has been studied. Obesity is known to be associated with systemic inflammatory reactions, insulin resistance, and oxidative stress. The pathways through which obesity could result in hypertensive disorder include increased oxidative stress, increased sympathetic tone, and increased expression of angiotensinogen [22]. Insulin resistance, on the other hand, is linked to reduced cytotrophoblast migration and consequent placental ischemia [23].

For pregnant women, maternal obesity, maternal overweight, and even a BMI increase within the normal range may indicate an increased risk of maternal and fetal morbidities, including preeclampsia. Accordingly, obese or overweight pregnant women should be advised to lose weight through diet control, a moderate amount of physical activity, and lifestyle modification.

### 3.6. Other Maternal Conditions

One of the most important risk factors for preeclampsia is a history of preeclampsia in a previous pregnancy. Women with preeclampsia in their first pregnancies have a notably higher risk of developing it again in their second pregnancies. Many cohort studies have demonstrated this phenomenon. [ref. Risk factors for pre-eclampsia at antenatal booking: systematic review of controlled studies]. Aside from her own medical history, a woman’s family history of preeclampsia should warrant special concerns as well, as a positive family history is also a powerful indicator of preeclampsia at all stages of the pregnancy course [24,25].

Multifetal gestation is associated with a three- to four-fold increased risk for preeclampsia. This may be more related to the gestation itself because multifetal gestation imposes a greater burden on the cardiovascular system. Individuals with multifetal gestations are frequently excluded from general studies, as they are often viewed as a special population. For these women who are diagnosed with preeclampsia, it may be confusing whether some of the adverse outcomes such as preterm deliveries are related to preeclampsia or multifetal gestation per se. Nevertheless, the fact that women with multifetal gestations are more prone to preeclampsia and serious complications should raise more attention, and allow an opportunity for screening, prevention, or early intervention [26].

Preeclamptic patients with pre-pregnancy chronic hypertension are classified to have “superimposed preeclampsia”. Chronic hypertension accounts for approximately 4% of pregnancies and is often associated with adverse gestational outcomes such as preeclampsia, preterm delivery, intrauterine fetal growth restriction, and placenta abruption. Approximately 20% of these patients eventually develop preeclampsia and tend to do so even earlier than the normotensive population. Poorer pregnancy outcomes are observed concurrently [27].

Both pre-existing type I and type II diabetes mellitus have been shown to possess a higher risk of preeclampsia. Statistically, 10–20% of diabetic women develop preeclampsia during pregnancy, which is a significantly higher percentage compared to their non-diabetic counterparts. On the other hand, gestational diabetes mellitus (GDM) is also considered to be an independent risk factor for preeclampsia by some researchers, while more investigations are required to determine whether GDM and preeclampsia share a common etiologic pathway [28].

While preeclamptic patients have an increased long-term risk of developing an end-stage renal disease, the renal disease itself may also serve as a risk factor for preeclampsia. To be more specific, microalbuminuria, diabetic nephropathy, and chronic kidney disease may predispose to preeclampsia [29]. This may be related to impaired glycocalyx integrity and alterations in the complement and renin-angiotensin-aldosterone systems [30].

Other than the aforementioned risk factors, mostly involving but not limited to pre-pregnancy health conditions, there are numerous other risk factors that could be mentioned. Aside from pre-existing hypertension, diabetes mellitus, and renal disease, particular medical conditions including autoimmune disease, periodontal disease, and antidepressant exposure have all been proven to play a part in this “disease of theories”. [31,32,33].

### 3.7. Socioeconomic Burden

Preeclampsia is one of the top causes of adverse maternal and fetal outcomes globally, and hence a short-term special medical care program is often required to take care of preeclamptic patients. This makes it not only a health-related issue but also a socioeconomic one, as greater manpower or resource consumption and extra spending in the healthcare system would be inevitable. Yet, few studies have aimed to make estimates of the potentially enormous socioeconomic burden related to preeclampsia. Relevant studies have made limited conclusions to date.

Jing Hao et al. conducted a retrospective study to investigate the economic burden of preeclampsia using data from the United States. Three cohorts were defined in this study: Women who had uncomplicated pregnancies until term, women with hypertension but not preeclampsia, and women diagnosed with preeclampsia. The maternal and infant costs were estimated from GA 20 weeks until 6 weeks postpartum for the former and 12 months post-delivery for the latter. The mean care cost of the preeclamptic group was $41,790 USD, which was significantly higher than the uncomplicated group ($13,187 USD) and the group with hypertension but without preeclampsia ($24,182 USD). The cost difference was largely dependent on the infant costs [34].

Another retrospective study focused on a similar issue in the United States was conducted by Warren et al. The maternal costs were estimated from 6 months before birth and 12 months afterward, while the infant costs were calculated until 12 months of age. The results suggested an estimated increased cost of $6583 USD per birth in the maternal model. On the other hand, the increased cost of the infant model was substantially influenced by the gestational age at birth. Costs devoted to the infant accounted for 26% of total healthcare costs at term delivery, and a tremendously increased percentage of 91% with deliveries at GA < 28 weeks. As a result of preeclampsia and preterm deliveries, these high costs were more closely related to adverse fetal or infant outcomes, including intraventricular hemorrhage, bronchopulmonary dysplasia, periventricular leukomalacia, and infant death [35].

An Irish study using data from the SCOPE (Screening for Pregnancy End Points) study disclosed a doubling average cost (5243 EUR) in preeclampsia-complicated pregnancies. The study included data from the initial antepartum visit until 12 months postpartum and drew the conclusion that these costs were primarily related to postpartum care, followed by antepartum and peripartum care, respectively. The increased medical costs were related to more and higher-level health services including antepartum examinations, more maternal hospitalization spending, longer infantile NICU stays, etc. [36].

Despite the lack of abundant research, present economic studies on preeclampsia in different parts of the world seem to have reached the consensus that preeclamptic pregnancies are strongly associated with significantly higher medical costs for both the mother and her baby. The maternal costs are related to the extra utility of the healthcare system, more resources used during hospitalization, and likely more surgical spending due to an elevated rate of cesarean deliveries. The infant costs also contribute to a large percentage of the expenses as the babies are prone to preterm deliveries and relevant or causative adverse events. Although studies cannot reflect the accurate amount of preeclampsia-related healthcare costs in reality, there is no doubt that preeclampsia imposes a considerable financial burden on our societies. It is important for healthcare providers and policy-makers to recognize this phenomenon and allocate enough economic budgets, medical, and social resources accordingly.

## 4. Pathophysiology of Preeclampsia

### 4.1. Brief Summary

Preeclampsia has been termed a “disease of theories” by some as numerous studies have aimed to propose different concepts to explore its complex etiology and pathophysiology. Previous findings have suggested that the triggers of preeclampsia include placental factors and other predisposing maternal factors. The mechanisms of early- and late-onset preeclampsia may not be completely the same. Based on the current understanding of preeclampsia, the revised “two-stage model” has become one of the most widely accepted theories regarding its formation.

The classical two-stage model was first described in 1991, innovatively introducing the idea that preeclampsia should be viewed as a trophoblastic disease rather than merely a hypertensive disorder. In this model, the first stage of preeclampsia is described as the “placental stage”, in which deficient remodeling of spiral arteries results in impaired placental perfusion and placental ischemia. As the disease progresses with time and clinical maternal syndrome develops, it reaches the second stage [37]. The clinical manifestations of preeclampsia will be further discussed in the next section.

**Figure 3:**
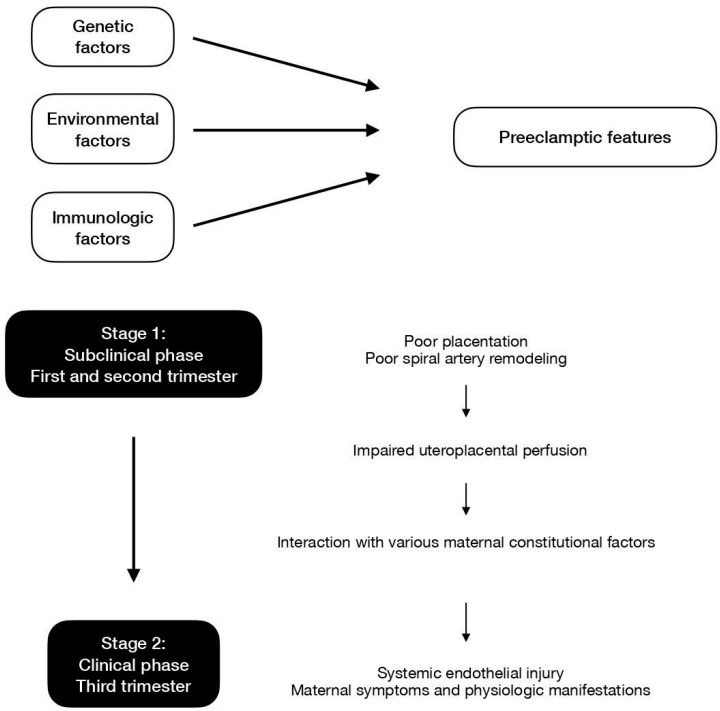
Contributing factors and the two-stage models of preeclampsia.

Ever since the initial proposal of the two-stage theory, ongoing research has expanded and refined our knowledge of the development of preeclampsia. Stage 1 is focused on the revised idea of impaired uteroplacental perfusion with or without poor placentation and subsequent spiral artery insufficiency. Stage 2 surrounds the concept that general endothelial dysfunction and vascular inflammation would lead to a systemic clinical response. Figure 3 displays the contributing factors and the two-stage models of preeclampsia.

### 4.2. Stage 1

In the current revised model, stage 1 is initiated when reduced placental perfusion develops. Poor placentation and the resultant deficient spiral artery remodeling in the intervillous space is one of the causes for this phase but may not be the sole mechanism. In addition to the impairment of placental perfusion, maternal factors are essential so as to result in the development of systemic maternal pathophysiological changes [37]. Stage 1 usually occurs in the first trimester, at the period of time when the deep invasion of the extravillous trophoblast (EVT) takes place. The migration of EVT cells into the decidua leads to the remodeling of maternal spiral arteries, which is a key element to uteroplacental perfusion and fetal blood supply. The process may initiate as early as before GA 8 weeks, while the establishment of uteroplacental circulation is completed at approximately GA 12 weeks. Hence, it is believed that stage 1 takes place before GA 12 to 20 weeks [38].

The differentiation and invasion of EVTs are regulated by various factors including cytokines, growth factors, chemokines, cell adhesion molecules, placental oxygen tension, extracellular matrix (ECM)-degrading enzymes, and membrane-bound cell surface peptidases. These factors are either directly or indirectly related to the differentiation and decidual invasion of EVT cells and may serve as markers for the first stage of preeclampsia formation [38]. When defective trophoblast invasion and insufficient transformation of the maternal uterine vasculature emerge, decreased maternal uterine blood flow follows, which may be detected and quantified by uterine artery Doppler studies. Persistent high vessel resistance in early pregnancy may suggest that the aforementioned phenomenon has occurred. Existing studies have demonstrated that the placental endothelial cells in women with high-resistance uterine arteries are more sensitive to TNFα and thus are more susceptible to cell injury and apoptosis.

In the normal process of trophoblast invasion, the resistance of uterine artery blood flow decreases, and the uterine artery blood flow increases in the term. The placenta is typically developed in the first trimester. If a relatively hypoxic environment is noted, the latter placental tissues may exhibit an altered balance of antioxidant enzyme activity. However, the histopathology findings of the placenta are nonspecific and not limited to preeclamptic pregnancies. These changes to the placenta can also be induced by other microscopic insults or toxins [39].

### 4.3. Stage 2

Stage 2 features the scenario where impaired uteroplacental perfusion interacts with other various maternal constitutional factors. Pathophysiological changes in the liver, kidney, and cardiovascular system are compatible with the concept of insufficient blood supply. Systemic endothelial dysfunction and injury are possible explanations for the maternal clinical manifestations and have been proven to be present in preeclamptic women.

One important issue that attracts interest is how the first stage links to or leads to the second stage. The clinical value of discovering the answer lies in the fact that it may shed light on a way to “prevent” the formation of preeclampsia, which will be further discussed in the article. One proposal suggests that microparticle particles produced during syncytiotrophoblast apoptosis may directly or indirectly result in endothelial dysfunction. An increased amount and concentration of inflammatory cells and substances have been found in women with preeclampsia, and they could potentially alter the systemic endothelial function. The renin-angiotensin system may also play a role in the process. In addition, some recent findings have suggested that vascular endothelial growth factor (VEGF) and placental growth factor (sFlt-1) could be involved in the linkage as well. Moreover, oxidative stress accumulated during the process may provide another possible explanation [40]. Table 4 lists the possible pathways and explanations of mechanisms underlying preeclampsia.

Various factors contribute to the regulation of artery compliance during pregnancy. A failure of maternal vascular adaptation can cause hypertensive disorders such as preeclampsia. Some circulating cytokines and growth factors at abnormal levels may inhibit normal calcium signaling events, thereby damaging cell-to-cell contacts of the endothelium and leading to endothelial dysfunction. Important markers include endothelin-1 (ET-1), interleukin-8 (IL-8), ELAM, and the endothelial leukocyte adhesion molecule-1 [41]. There is also sound evidence of decreased production or bioavailability of nitric oxide (NO)—a stimulant of smooth muscle relaxation—in preeclamptic pregnancies [42]. Other potential influential vasodilators include prostacyclin (PGI2) and the endothelium-derived hyperpolarizing factor (EDHF) [41].

As mentioned above, the dysregulation of the renin-angiotensin system (RAS) may participate in the pathogenesis of preeclampsia. In 2007, Florian Herse et al. published a study that included preeclamptic and non-preeclamptic women who had undergone cesarean deliveries. Genetic characteristics and histopathological results of the maternal and placental tissues of the participants were investigated. A 4-fold increase in the angiotensin II type 1 (AT1) receptor in the decidua was found in preeclamptic pregnancies. Increases in corresponding gene and protein expression were also confirmed by RT-PCR and immunohistochemistry studies. Circulating agonistic autoantibodies (AAs) targeting the AT1 receptor have been described previously, with the ability to cross the placenta and enter fetal circulation. AT1-AAs could induce calcium signaling and initiate events that would later lead to preeclampsia [43]. Roxanna A. Irani et al. published a study with similar findings in 2010. Animal experiments showed that pregnant mice with AT1-AA injections developed preeclamptic features and also had increased levels of antiangiogenic factors such as soluble fms-like tyrosine kinase 1 (sFlt-1) and endoglin. Additionally, AT1-AA might be associated with increased TNF-α, indirectly causing damage to the endothelium and end organs [44].

Oxidative stress describes the imbalance between the formation of oxidative reactive species (ROS) and the antioxidant capacity of the body [45]. A causal role of oxidative stress in hypertension has been demonstrated in previous research, with multiple possible pathogenic pathways including the alteration of NO bioavailability or signaling. A reduction of oxidative stress has been observed in hypertensive cases who received antihypertensive treatment [46]. Oxidative stress in the healthy placenta may be important for its organogenesis, but excess levels in the impaired placenta would lead to increased circulating placenta debris, damaging the maternal endothelial cells in the term. As a major source of ROS production, the mitochondria have been found to be swollen in the trophoblasts of preeclamptic animal models, which plays a crucial role in cell apoptosis. Altogether, any errors in the maintenance of the oxygen pressure may bring about placental diseases and maternal complications, such as preeclampsia [46].

### 4.4. Limitations of the Placenta Model

Even though the two-stage theory is the mainstream explanation of the origin of preeclampsia, some argue that further evaluation is needed to determine the causative relation between trophoblast development and spiral artery transformation. For instance, previous case reports have pointed out similar findings of the uterine artery Doppler waveforms in extra-uterine pregnancies, suggesting that the resistance of uterine artery blood flow may not accurately reflect the consequences of trophoblast invasion [47].

Some have suggested that the result of Doppler studies may be a reflection of systemic vascular resistance changes but not on the uterine artery itself. The argument is based on the paradox that a “de-transformation” of spiral arteries does not occur when the vascular resistance of the uterine artery is noted in the third trimester [48]. In the meantime, while it is fairly certain that impaired uteroplacental perfusion is associated with subsequent endothelial dysfunction, almost all the supporting evidence of different hypotheses of its linkage raises some challenges. To date, it is believed that many potential mechanisms underlie preeclampsia, and the disease is caused by complicated interactions between maternal and environmental factors, and potentially more than that. The incomplete understanding of its pathogenesis continues to provoke further research.

## 5. Systemic Manifestations of Preeclampsia

**Figure 4:**
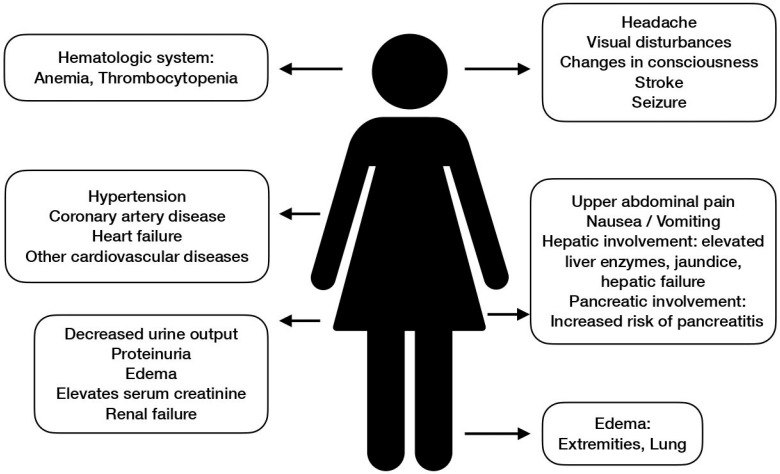
Systemic manifestations of preeclampsia.

Preeclampsia is a systemic disorder that may present with various symptoms and signs. The manifestation of preeclampsia is widely perceived to be centered around hypertension and proteinuria, but clinical presentations could be variable in essence. Different organs and systems could all be influenced by preeclampsia. Systemic manifestations of preeclampsia are shown in Figure 4.

According to the two-stage theory, preeclampsia proceeds into the clinical stage once the systemic vascular response and inflammation have taken place as a result of endothelial dysfunction. This aptly explains why preeclampsia is a global syndrome as the endothelium is distributed all over the body. The most famous affected organs and systems include the central nervous system, cardiovascular system, liver, and kidney.

### 5.1. Central Nervous System (CNS)

The brain is a vital organ that requires approximately 20% of available oxygen to maintain normal function. In most physiological conditions, cerebral blood flow (CBF) has sufficient capability to autoregulate and remains rather stable to cope with its high metabolic demand. However, once brain injury to a certain extent occurs, sequelae including acute severe hypertension, the loss of myogenic tone of the vascular smooth muscle, and uncontrolled vasoconstriction may lead to the failure of autoregulation. As they key to the delicate homeostasis of the brain environment, both hypoperfusion and hyperperfusion may break the balance and bring great harm. Insufficient CBF could lead to ischemic brain injury and ischemic stroke. Hyperperfusion, on the other hand, may disrupt the blood–brain barrier (BBB) and cause edema formation, which is one of the classic findings in preeclamptic and eclamptic patients [49,50].

CBF could be assessed in patients with preeclampsia via transcranial Doppler imaging. The middle cerebral artery is often chosen to be the target of examinations. Perhaps somewhat surprisingly, the cerebral flow index (CFI) appears to be normal in most women with preeclampsia. However, cerebral perfusion pressure (CPP) exhibits greater elevation in preeclamptic women and may serve as the key to brain injury among these women [51]. Brain damage has been demonstrated in autopsies and image studies [52]. It has been proven in some studies that elevated CPP corresponds to hypertension, and antihypertensive treatment that decreases CPP lowers the rate of cerebral complications in these patients [53].

CNS manifestations that are suggestive of severe disease status are headaches, visual disturbances, changes in consciousness, and seizures. The spectrum is coined “preeclamptic encephalopathy” [53]. Once a seizure takes place, the impression of eclampsia is almost certain after the exclusion of other previously known neurological conditions that may also lead to convulsion events. Eclampsia is one of the most serious forms of preeclampsia and is highly related to obstetric morbidities and mortalities. Management for eclampsia is similar to those for any form of severe preeclampsia, and most patients recover well without neurological sequelae [54].

Another frequent neurological finding is posterior reversible encephalopathy syndrome (PRES). PRES is a result of hypoxia and vasogenic edema of the brain that is often related to acute uncontrolled hypertension or systemic endothelial dysfunction. The syndrome progresses in a rather rapid manner but also resolves rapidly with a good prognosis once the trigger is withdrawn [55]. PRES is high among pregnant women with severe preeclampsia or eclampsia, and usually indicates a better prognosis than PRES in non-pregnant women or is associated with other causes [56].

Stroke—or a cerebrovascular accident—refers to a brain attack when impairment of part of the CBF or a burst in a brain blood vessel occurs. Strongly related to hypertensive disorders, cerebrovascular events are yet another complication that is significantly linked to preeclampsia. Although uncommon in pregnancy, it shares similar disease pathways and risk factors with strokes that take place in non-pregnant patients, and thus is indicative of an increased long-term risk for stroke events [57].

### 5.2. Cardiovascular Systems

Preeclampsia has been classified as one independent gender-specific risk factor for cardiovascular events by the American Heart Association (AHA). Studies have proven that women with gestational hypertensive disorders carry a 2- to 4-fold risk for cardiovascular diseases [58]. In fact, preeclampsia and cardiovascular diseases share many predisposing factors such as elevated blood pressure and increased BMI. The disease spectrum includes coronary heart disease, heart failure, and cardiovascular disease death, and the influences may be life-long [59].

The long-term cardiovascular sequelae not only affect the mother but have also proven to bring hazards to her children at the same time. Although the establishment of a dependency relationship is difficult, many studies have shown an increased rate of congenital heart disease and future cardiovascular morbidities for offspring. Some scholars believe, however, that the influences of cardiovascular risks on offspring are limited to term infants or cases with early preeclampsia [60].

The complex multifactorial nature of preeclampsia and cardiovascular diseases makes it hard to make a straightforward ascription of the latter to the former. There is also a lack of a standardized protocol for cardiovascular prevention. Nevertheless, medical staff and preeclamptic patients should keep in mind the importance of continuous screening and early intervention of cardiovascular diseases. Monitoring of the body weight, blood pressure, lipid level, and lifestyle should be performed every five years until the age of 50 when women would qualify for most other international regular cardiovascular risk assessment guidelines [61].

### 5.3. Liver

Preeclampsia-related liver disease is frequently seen in the third trimester. Liver involvement is rare but indicative of severe disease extent. The most notorious example is H(Hemolysis)EL(Elevated Liver Enzymes)LP(Low Platelet Count) syndrome, which is a variant of severe preeclampsia. According to the diagnostic criteria of Tennessee Classification and Mississippi Classification, an elevated liver enzyme is usually defined by an elevated AST or ALT ≥ 70 U/L, although blood tests often reveal a level ≥ 500 U/L. Thrombotic microangiopathy serves as one of the possible explanations, while periportal hemorrhage and necrosis have been observed in histopathology studies. As rare as it may be, the condition could lead to hepatic rupture [62]. Women treated with corticosteroids exhibit overall improved laboratory results including liver function tests. Administration after delivery helps to avoid a rebound and further complications. Nevertheless, the natural course of HELLP could not be altered by corticosteroids [63].

Other liver diseases associated with preeclampsia include acute fatty liver of pregnancy (AFLP), hepatic infarction, and rupture. In cases of AFLP, laboratory abnormalities include elevated liver enzymes, prolonged prothrombin time and partial thromboplastin time, and increased bilirubin levels. Other typical clinical symptoms comprise central nervous system involvements such as headache and consciousness disturbances, jaundice, and gastrointestinal symptoms including anorexia, abdominal discomfort, nausea, and vomiting. If the expression of long-chain 3-hydroxyacyl-CoA dehydrogenase is not evident, the prognosis is usually good [64].

Hepatic complications in pregnancy are rare but could be fatal. They are more likely to be found in preeclamptic or eclamptic cases and indicate severe disease status. Hence, prompt termination of pregnancy or delivery is often indicated. Liver transplantation may be considered in patients with a grave prognosis [63].

### 5.4. Kidney

The imbalance of the renin-angiotensin aldosterone system (RAAS) along with the imbalance between proangiogenic and anti-angiogenic factors may explain the relationship between preeclampsia and renal impairment. Similar to cardiovascular risks, preeclampsia shares common predisposing factors with renal risks and confers a higher risk of chronic kidney diseases later in life [65].

The activation of RAAS is normal during pregnancy, which results in a volume increase. However, excessive activation possibly related to sFlt-1 and AT1-AAs—as may be seen in preeclamptic subjects—could lead to preeclampsia or preeclampsia-like syndrome. Once the delicate balance is disrupted, hypertension and renal involvements may be seen [66]. Thrombotic microangiopathies in renal cells have been observed in histopathology studies, suggesting glomerular injury in preeclamptic patients [67].

When acute kidney injury occurs, an abrupt increase in serum creatinine and a decrease in urine output could be detected. However, both the glomerular filtration rate and serum creatinine level are not perfectly reliable markers during pregnancy, as physiological changes allow an increase in the former and a reduction in the latter. The diagnosis may rely on other clinical manifestations such as oliguria, proteinuria, and edema, and is thereby delayed in some cases [68].

The condition may be life threatening, but also tends to regress rapidly in the postpartum period. Nonetheless, it still warrants concern for screening for later renal diseases. There is a significant association between preeclampsia—the early-onset subgroup in particular—and future chronic kidney diseases, hypertensive diseases, and glomerular or proteinuric diseases. For preeclamptic women, a 10- to 12-fold increase in end-stage renal disease has been proposed in existing statistical analyses. Hence, further screening for kidney diseases years after pregnancy should be implemented [69].

### 5.5. Other Targets

Preeclampsia is a global disorder that may present with symptoms and pathologic findings all over the body. Aside from the vital organs, for example, the hematologic system is another commonly affected target. In a study conducted by Neelam Jhajharia et al., lower hemoglobin and platelets were found in these patients, while higher WBC and hematocrit were observed [70]. Similar findings could also be found in other studies [71,72]. Some parameters may vary from study to study, but a trend of decreased hemoglobin and platelet levels is almost always observed in data analyses. Marked thrombocytopenia signifies a severe disease form, as manifested in HELLP syndrome.

Gastrointestinal involvements are common in preeclamptic patients. Symptoms of nausea and vomiting are frequently experienced by them, and some women complain of indigestion. The more devastating complications include hepatic involvement as described earlier, and pancreatic involvement, namely referring to the increased risk of pancreatitis and necrosis of the pancreas [73].

Another classic clinical manifestation of preeclampsia is edema. It is worth noting that edema is not essential to the diagnosis of preeclampsia and is often observed in normal pregnancies as a result of the increase in body fluids. General swelling due to water and salt retention is especially prominent in preeclampsia due to elevated blood pressure and endothelial injury causing extravasation from the vessels. As rare as it may be, one of the most severe presentations of fluid overload is pulmonary edema, which has been reported in cases of severe preeclampsia [74].

## 6. Prediction and Prevention of Preeclampsia

The potential consequences of preeclampsia pose great threat and harm to mothers and their children, the medical system, and society worldwide. To prevent adverse outcomes, various strategies have been invented and studied, including diet control, exercise, and medication. Among them, the administration of low-dose aspirin has been proven to be one of the most effective ways to prevent the development and progression of preeclampsia.

In order to apply preventive methods in a cost-effective manner, a precise prediction model and timing would be required. According to the two-stage theory of its pathophysiology, the first stage of preeclampsia typically takes place in the first trimester, when inadequate trophoblast invasion leads to abnormal placentation and subsequent uteroplacental insufficiency. During this process, the patient is usually in her subclinical phase, which allows a window for screening and prevention.

### 6.1. Prediction Models

Apparently, a good prediction tool would provide many benefits to the early prevention and intervention of preeclampsia. Different professional organizations have thus far proposed their own prediction models based on the currently acknowledged risk factors.

An expert review written by Piya Chaemsaithong et al. made a detailed comparison between some of the most widely accepted prediction models (Table 5). Risk factors such as a history of preeclampsia in previous pregnancies, chronic hypertension, autoimmune diseases, renal diseases, diabetes mellitus, and multifetal gestation are included in almost all the prediction models and are primarily considered to be “high” risk factors if the models made a segmentation between “high” risk and “moderate” risk factors. Other risk factors that are taken into consideration include nulliparity, advanced maternal age, maternal obesity, and family history, among others. Some are classified as “moderate” risk factors. A previous medical record and chronic hypertension are considered to be the two most important contributory risk factors [5].

Most prediction models have either low detection rates or high false-positive rates, however, and are insufficient for precise prediction. An alternative is to use the Bayes theorem and take the individual maternal history and characteristics into consideration. This competing model allows a more patient-specific and dynamic approach and is used by the Fetal Medicine Foundation (FMF) and is the only one that has undergone extensive internal and external validations. In addition to the checklist for risk factors, other maternal factors including the mean arterial pressure (MAP), uterine artery pulsatility index (UtA-PI), and serum pregnancy-associated plasma protein A (PAPP-A) are also taken into account [5,75]. The best timing of preeclampsia risk screening is around GA 11 to 13 weeks. As soon as the result is revealed, early prevention could be initiated if a high risk for preeclampsia is suspected [76].

A systematic review examined the performance of soluble fms-like tyrosine kinase-1 (sFlt-1), the placental growth factor (PlGF), and the sFlt-1/PlGF ratio in predicting adverse outcomes in women with preeclampsia. The literature search identified 33 eligible studies (n = 9426). Due to significant heterogeneity between studies, few studies (n = 4–8) were included in the final meta-analysis component. Nonetheless, both PlGF and the sFlt-1/PlGF ratio demonstrated areas ROC values between 0.68 and 0.87 for the prediction of composite adverse maternal and perinatal outcomes, preterm birth, and fetal growth restriction. Conclusively, PlGF and the sFlt-1/PlGF ratio show prognostic promise for adverse outcomes in preeclampsia, but study heterogeneity limits their clinical utility [77].

Currently, prediction models for gestational hypertension and preeclampsia have been developed with data and assumptions from developed countries. A review aimed to identify and assess the methodological quality of prediction models for gestational hypertension and pre-eclampsia with reference to their application in low-resource settings. The review retrieved 40 eligible articles and revealed 77% of all the prediction models’ combined biomarkers with maternal clinical characteristics. The biomarkers used as predictors in most models were PAPP-A and PlGF. Only five studies were conducted in low- and middle-income countries. Therefore, the review concluded that prediction models using maternal characteristics, with good discrimination and calibration, should be externally validated for use in low- and middle-income countries where biomarker assays are not routinely available [78].

### 6.2. Possible Preventative Measures

An article written by Sammya Bezerra Maia and Holanda Moura et al. offered a perspective on the potential preventative measures by classifying them as primary, secondary, or tertiary preventions. Primary prevention is unlikely to be successful since the concept is centered around the avoidance of pregnancy in high-risk populations and lifestyle modification in the whole population in order to decrease the incidence of preeclampsia. Secondary prevention focuses on the interruption of the pathogenic process before its development and is the main target of investigations. Tertiary prevention does not aim to prevent preeclampsia itself, but rather prevent its further complications. Aside from the aforementioned section on aspirin prevention, lifestyle management, nutritional supplementation, and antenatal surveillance may aid primary and secondary prevention. Accordingly, rest, exercise, diet modification such as a low-salt diet, and antioxidant use have all been suggested. Unfortunately, none of them have been proven to be effective [79]. In contrast, another systematic review solicited 28 RCTs studying the effects of various factors such as anticoagulants (heparin, enoxaparin, dalteparin, and nadroparin), aspirin, paravastatin, nitric oxide, yoga, micronutrients such as L-arginine, folic acid, vitamin E and C, phytonutrients, lycopene, and vitamin D alone or in combination with calcium. The results of this review showed that low-molecular-weight heparin, enoxaparin, yoga, L-arginine, folic acid, and vitamin D prevented preeclampsia alone or combined with calcium [80].

### 6.3. Low-Dose Aspirin

In fact, for women perceived as high-risk individuals for developing preeclampsia, the administration of low-dose aspirin on a daily basis since early pregnancy has proven to be the most effective way to prevent preeclampsia.

Aspirin is one of the oldest medications still in use to date. It is widely applied as an antithrombotic drug due to its effect on platelet inactivation. The mechanism of platelet inactivation relies on COX-1 inhibition, which blocks TXA2 synthesis. It is usually administered in a low dose (75–81 mg/day), which is sufficient for TXA2 but not PGI2 inhibition. The application of aspirin is primarily related to secondary prevention for cardiovascular diseases, while its use in primary prevention remains somewhat controversial. Another common use of aspirin is anticoagulant treatment in neurological diseases such as transient ischemic attack (TIA) or stroke. A recent history or increased risk for gastrointestinal bleeding, intracerebral bleeding, and other adverse events is worth noting, and aspirin should only be administered when the benefits outweigh the risks—primarily related to the increased bleeding tendency [76].

Ever since the first publication of a case report suggesting the role of aspirin in preeclampsia prevention in 1978, numerous studies have aimed to quantify the effects of aspirin on preventing preeclampsia but without a consensus owing to the heterogeneity of the study groups. Meanwhile, meta-analyses have suggested prophylactic aspirin use, which is best started before GA 16 weeks and ahead of the completion of placentation. Later, the ASPRE trial confirmed the effect of aspirin on early-onset preeclampsia. With good compliance, aspirin prophylaxis could reach 76–90% effect size [79].

A meta-analysis including a total of 18,907 participants in eight trials reported that the administration of aspirin was associated with a reduction in the risk of preterm preeclampsia (relative risk: 0.62; 95% confidence interval: 0.45–0.87), but there was no significant effect on term preeclampsia (relative risk: 0.92; 95% confidence interval: 0.70–1.21). The reduction in preterm preeclampsia was confined to the subgroup in which aspirin was initiated at ≤16 weeks of gestation and at a daily dose of ≥100 mg (relative risk: 0.33; 95% confidence interval: 0.19–0.57). Thus, aspirin can reduce the risk of preterm preeclampsia rather than term preeclampsia, and only when it is initiated at ≤16 weeks of gestation and at a daily dose of ≥100 mg [81].

Many organizations have proposed their own guidelines regarding the dosage and timing of aspirin use. Table 6 presents the current recommendations for the administration of low-dose aspirin in women at risk of future preeclampsia. While some differences exist, most agree on a daily dosage between 60 and 150 mg per day, and some have a precise dosage of 81 mg per day for low-dose aspirin tablets in the U.S. Administration is recommended late in the first trimester and could be initiated as early as GA 12 weeks and before GA 16 weeks. Low-dose aspirin is usually prescribed until the late preterm period. Bleeding disorders are uncommon, while some women might experience a certain degree of gastrointestinal discomfort [79,82].

Low-dose aspirin use for preeclampsia prevention has been proven to be cost-effective and safe in pregnancy. Therefore, when the prediction model suggests a high risk of preeclampsia or when a mother carries some risk factors, low-dose aspirin should be initiated if no contraindications are identified.

## 7. Management of Preeclampsia

Table 7 is a summary of the current management practices of preeclampsia.

### 7.1. Antihypertensive Treatment

Elevated blood pressure is essential to the diagnosis of preeclampsia and is associated with increased cardiac, vascular, and neurological risks. Therefore, antihypertensive medication should be administered for the control of blood pressure.

Some studies divide gestational hypertension into “severe” and “non-severe” groups, typically setting 160 mmHg as the cutoff point for systemic blood pressure (SBP). While the choice of antihypertensive regimen may be designed in an individualized manner, most clinicians agree with the initiation of antihypertensive therapy when the SBP exceeds 140 or 160 mmHg or when the diastolic blood pressure (DBP) reaches above 100 mmHg. The treatment target also differs between different guidelines; some set no specific treatment target, while some target an SBP below 110 mmHg or a DBP less than 80 to 90 mmHg [83].

The majority of common antihypertensive medications are contraindicated during pregnancy, so the choice of drugs is rather limited. Currently, almost all the approved medications belong to class C, including labetalol, hydralazine, nifedipine, methyldopa (class B), diazoxide, and the relatively contraindicated nitroprusside. The former three are used more frequently even though the FDA has not approved the usage of nifedipine in hypertension management [83].

#### 7.1.1. Labetalol

Labetalol could be administered in the intravenous or oral form, while the intravenous form is more often used in hypertensive emergencies or grave conditions. It is a combined alpha- and beta-adrenoceptor-blocking agent with more potency on the beta receptor. It is widely used as an antihypertensive agent and has the advantage of exerting a minimal effect on heart rate and cardiac output. Side effects and adverse events are primarily related to the influence on the RAAS and respiratory system. Therefore, other alternatives should be contemplated for patients with asthma [84].

When given in preeclamptic pregnancies, labetalol also decreases proteinuria and perinatal deaths. No other antihypertensive medications have proven to produce similar effects [85]. In non-severe cases, the oral dosage of 200 to 1200 mg per day can be divided into two to three doses, depending on the individual condition [83]. On the other hand, an intravenous bolus of 20 mg labetalol is indicated in severe cases and could be followed by a double dose in ten minutes [86].

#### 7.1.2. Hydralazine

Hydralazine is another popular medication of choice, which also comes in intravenous and oral forms similar to labetalol. It lowers blood pressure by acting as a direct arteriole vasodilator. Headache, flushing, chest discomfort, and gastrointestinal upset have been reported with hydralazine use. It is also known to be associated with drug-induced lupus syndrome [87]. However, drug toxicity is uncommon. The drug is fairly safe except for contraindication in women with coronary artery disease since the increased cardiac output and oxygen demand may be hazardous [87]. Its effects have been proven, but it may be less efficacious than other antihypertensive drugs such as labetalol and nifedipine and is perceived by some as a second-line choice instead of a first-line option [85]. In regular oral use for blood pressure control, the recommended regimen is to start with 10 mg four times per day with gradual adjustment. The maintenance dosage could be as much as 50 mg four times per day and still has room for titration as long as it does not exceed the daily maximum dosage of 300 mg [87].

#### 7.1.3. Nifedipine

Nifedipine is a safe and effective oral drug for lowering blood pressure in preeclamptic patients. The advantages include its relatively low cost and wider accessibility [88]. This medication functions as a calcium channel blocker with a rapid vasodilating effect but is associated with few adverse events and a low risk of hypotension [89]. Headache, flushing, and palpitations are the most frequent complaints encountered [90]. Its simultaneous relaxing effect on the myometrium also makes it a tocolytic drug commonly used to avoid preterm delivery [90]. It is usually initiated with a 10 mg dose and could be repeated later [86].

### 7.2. Magnesium Sulfate

Magnesium sulfate is used extensively for the prevention of seizures in preeclampsia and recurrent seizures in eclampsia for a lengthy period of time. Compared to placebo and other anticonvulsants such as phenytoin and diazepam, magnesium sulfate has proven to be more effective with fewer side effects. Although the mechanism is not fully understood, several possible explanations have been proposed [91].

Since the twentieth century, the action of magnesium sulfate used in preeclampsia has been studied. It is less likely related to antihypertensive effects as eclampsia does not necessarily take place in a hypertensive condition. Instead, magnesium sulfate may function as a calcium antagonist and inhibit acetylcholine-calcium-dependent release. While calcium may induce vasospasm and activate smooth muscle constriction, magnesium works in the opposite fashion. Since cerebral vasospasm is a common finding in preeclamptic seizures, this may serve as a plausible explanation to justify the use of magnesium sulfate. Another hypothesis suggests magnesium functions as a blocker of NMDA receptors, and thus prevents calcium influx [92,93].

In women with severe preeclampsia or eclampsia, magnesium could be given with an initial loading dose via the intravenous or intramuscular route, followed by a maintenance infusion. To reach the therapeutic serum concentration of 3.5 to 7 mEq/L (4.2 to 8.4 mg/dL), the recommended loading dose is 6 g intramuscularly, 2 to 4 g intravenously with a rate of 1 g/hour, or a combination of both. Different guidelines and studies may suggest a slight modification. Some minor side effects such as a warm sensation, flushing, nausea, and vomiting may be encountered within the therapeutic window. However, serious adverse events may occur if marked hypermagnesemia is noted. The loss of the normal patellar reflex may be seen when the serum concentration of magnesium reaches 8 to 10 mEq/L, and more devastating respiratory depression could result when the serum concentration of magnesium reaches or exceeds 13 mEq/L. Therefore, persistent monitoring of the neurological performance such as the presence of the patellar reflex, respiratory pattern, and urine output should be implemented to avoid magnesium toxicity and severe adverse events. If a dosing error or toxicity is noted, calcium gluconate could be administered as an antidote [94].

Overall, magnesium sulfate use in pregnant women is still considered to be safe as long as close surveillance is performed. Its effects on seizure prevention have been assuring, and associated morbidity and mortality rates are low.

### 7.3. Delivery and Termination of Pregnancy

Preeclampsia is a pregnancy-specific condition, which would require delivery or termination of pregnancy under certain circumstances. A dilemma between expectant management and delivery is sometimes faced, especially when the patient has not reached term pregnancy. Hence, the timing of delivery in preeclamptic patients has raised keen discussions.

Lucy C Chappell et al. conducted a randomized clinical trial on late-preterm preeclamptic patients to survey this issue. A total of 901 gravidas with preeclampsia from GA 34 weeks to less than GA 37 weeks were included and randomly allocated to expectant management or planned delivery evenly. Planned delivery was initiated within 48 h of randomization to allow corticosteroid use if needed, and labor induction was prioritized unless an indication for cesarean delivery existed. The maternal outcomes included severe hypertension, deficits, impairments of different organs, placenta abruption, and maternal death. The perinatal outcomes were a composite of NICU stays, neonatal deaths, and further neurological developments. The statistical findings suggested a significantly lower rate of maternal morbidities and mortalities but more adverse perinatal outcomes in the planned delivery group. The higher rate of adverse perinatal outcomes, however, was primarily related to NICU admissions due to preterm birth, and other neonatal outcomes were similar to those in the expectant group otherwise. The total maternal and neonatal medical costs were lower in the planned delivery group. Collectively, the results suggested planned delivery in women with late-preterm preeclampsia, but the risk of increased NICU admission, although not associated with further morbidities, should be informed and discussed with the patient [95].

A systematic review in 2017 solicited six articles regarding preterm preeclampsia and the timing of delivery. The subjects of discussion included both early-preterm and late-preterm women. The statistics suggested postponing delivery until GA 37 weeks for better fetal outcomes, and no severe maternal complications or fetal distress existed. However, delivery should be considered even with early preterm patients once severe maternal complications or impaired fetal well-being was noted. It is important to note that suggested delivery is not equivalent to an emergency delivery and should still allow a 24-h interval for preparation (e.g., corticosteroid use to promote fetal lung maturity) since an immediate delivery is often associated with greater risks. On the other hand, women who chose expectant management should receive close monitoring and medication if needed. For example, magnesium sulfate could be used to reduce the risk of eclampsia, and more recently, has been suggested as a neuroprotective medication for the fetus before GA 32 weeks in particular [96].

According to the most updated ACOG guidelines in 2018, the choice between expectant management and prompt delivery should be made based on gestational age, maternal condition, and fetal well-being. Those with reassuring antenatal testing, no signs of preterm labor, and no severe disease features make good candidates for expectant management until GA 37 weeks. There are no benefits to delaying delivery afterward. Conversely, those with potentially severe features and a worrisome fetal condition should be advised for immediate delivery. For patients with severe preeclampsia, early delivery indicates a trade-off between fetal benefits and maternal risks, and a thorough discussion between the medical team and the patient should be performed. Delivery should be considered at any time when the maternal or fetal condition deteriorates or becomes unstable regardless of the gestational age. A complete course of corticosteroid administration is not always necessary, especially when the woman has reached late preterm (GA ≥ 34 weeks) [97].

### 7.4. Fluid Management

Fluid management is important in women with preeclampsia because they are more prone to fluid overload, which could lead to pulmonary edema. However, research on an ideal fluid strategy has been limited to date. Present data fail to suggest an optimal regimen, in turn provoking future research [98]. Nevertheless, intravenous medication with fluids is almost inevitable for hospitalized patients and should therefore be administered with caution. For instance, some clinicians have recently suggested avoiding intravenous fluid preloads before epidural or spinal anesthesia [98].

### 7.5. Diet Management

Fl Diet and nutrient intake may impact the risks of preeclampsia, and some studies have aimed to seek the best policy for diet management. In 2022, BMJ published a review based on data from 2000 to 2021 regarding the effects of dietary factors, nutritional supplements, and maternal weight on preeclampsia. Some of the findings may be contradictory to public belief; for instance, a low-salt diet to prevent hypertension and antioxidant (e.g., Vit. C and E) supplements to relieve oxidative stress seem to be plausible ways to prevent preeclampsia, but the review fails to show enough evidence in reality. However, it is important to note that a low amount or lack of evidence does not imply that they are not helpful and should not be recommended. In fact, the dietary factors that have proven to reduce the risk of preeclampsia include maternal weight control, high fiber intake, probiotics use, calcium and vitamin D supplements, multivitamin and multimineral supplements, and the avoidance of a high-salt diet and raw food [99].

A cohort study conducted by Anum S. Minhas et al. suggested that a self-reported Mediterranean-style diet is associated with lower preeclampsia risks. A Mediterranean-style diet is rich in vegetables, fruits, and healthy fats, of which the coherence could be assessed with a food-frequency questionnaire, in which individuals answer the questions by recalling their eating habits regarding meat, seafood, vegetables, beans, fruits, oil, wine, sweetened beverages, and commercially baked foods. It has been previously proven to lower cardiovascular risks in the non-pregnant population, and this study further suggested that greater adherence is associated with >20% lower odds of developing preeclampsia in the pregnant population compared to women with less adherence [100].

### 7.6. Exercise

Many clinical and animal studies have identified significant or non-significant effects of exercise during pregnancy on the reduction of gestational hypertensive disorders including preeclampsia.

There are a few possible explanations in so for the results observed. To begin with, maternal exercise creates a transient hypoxic environment, which in turn promotes the compensatory proliferation of the trophoblastic, endothelial, and stromal cells of the placenta, and consequently leads to improved placentation. Secondly, exercise stimulates antioxidant pathways and increases the number of mitochondria, relieving much of the oxidative stress that is linked to preeclampsia. Thirdly, exercise has an anti-inflammatory effect, which allows the body to maintain a healthy immune reaction, thus reducing the abnormal immune response to the fetus that is witnessed in preeclamptic patients [101].

A review study in 2017 suggested that aerobic exercise is beneficial in pregnancy and should be encouraged. Whether or not aerobic exercise could reduce preeclampsia remained controversial in some studies, but overall, the review concluded that 30 to 60 min aerobic exercise two to seven times per week during pregnancy reduced the incidence of gestational hypertensive disorders and the rate of cesarean deliveries [102]. However, this may not be the perfect regimen for every pregnant woman. The pre-pregnancy physical activity levels and maternal condition should always be taken into consideration for physicians to offer the best advice on the frequency, intensity, type, and time of exercise [103].

### 7.7. Long-Term Follow Up

Last but not least, preeclampsia is a syndrome that develops before delivery, yet also demands extra healthcare in the long run. Long-term follow-up for potential complications is indicated since sequelae of the cardiovascular system, liver, and kidney could take place. Close surveillance for years is suggested, which requires alertness from a good medical team and good medical compliance from the patient herself.

## 8. Discussion

The true cellular and molecular mechanisms underlying preeclampsia remain largely unexplained, which are assumed to be a two-stage process of impaired uteroplacental perfusion with or without prior defective trophoblast invasion (stage 1), followed by general endothelial dysfunction and vascular inflammation that lead to systemic organ damages (stage 2). Although the causes of preeclampsia are multi-factorial and cannot be described in a simple way, the aforementioned theories may provide a reasonable explanation for the results observed in past studies. Nevertheless, the detailed etiology, pathophysiology, and effect of preeclampsia seem complicated and remain to be clarified.

As mentioned above, overweight including pre-pregnancy obesity and excessive weight gain during pregnancy predisposes women to the progression of preeclampsia. As a state of chronic inflammation, overweight will increase the risk of preeclampsia by means of activating macrophages, NK cells, and peripheral helper T cells within the placenta to produce inflammatory cytokines such as IL-6, IL-7, and TNF-α. Established on these findings and reasons, avoiding excessive weight gain before and during pregnancy, rather than merely using overweight as a predictor, may be the best strategy to prevent the occurrence of preeclampsia. Therefore, proper weight control for pregnant females can not only decrease the physical burden on the body but also reduce the risk of preeclampsia.

Well-recognized risk factors for preeclampsia include race, advanced maternal age, obesity, nulliparity, multi-fetal pregnancy, and co-existing medical disorders. These factors can serve as warnings or markers to label pregnant women who need enhanced surveillance of maternal and fetal well-being. For at-risk females, appropriate information, counseling, and suggestions should be provided to facilitate a timely intervention or specialty referral. For pregnancies complicated with preeclampsia, closer monitoring and antepartum surveillance including a Doppler ultrasound blood flow study, biophysical profile, non-stress test, and oxytocin challenge test can be arranged. If the results are unfavorable, early intervention and aggressive therapy should be considered. Moreover, affected females should have access to higher levels of obstetric units and neonatal institutes. Before, during, and after delivery, monitoring and preparation should be intensified for affected gravidas to avoid serious complications of preeclampsia. In response to the potential physiological effect and psychological impact, consultation and discussion are usually beneficial for females diagnosed with preeclampsia. For severe cases, delivery of the fetus and placenta is the ultimate solution to treat preeclampsia. However, the determination of the appropriate timing for delivery depends on the severity of maternal preeclampsia and the maturity of the fetus.

It remains not fully understood with regard to the molecular level and pathologic mechanism of preeclampsia and its associated treatment. More studies are still required to investigate the role of anticoagulant therapy (such as aspirin, as described above) in preeclampsia. To minimize the heterogeneity of research in the future, the standardization of several critical factors in preeclampsia and the related treatment should be carefully considered. Two of the important factors are the timing and intensity of screening and intervention, which have a remarkable impact on the therapeutic effects. Furthermore, the severity and outcome in the individuals diagnosed with preeclampsia need standardization. Moreover, a larger sample size is also required to draw a reliable conclusion and to improve the reproducibility of the study result.

## 9. Conclusions

Preeclampsia accounts for one of the most common documented gestational complications, with a prevalence of approximately 2 to 15% of all pregnancies. It is life-threatening for both the mother and the fetus, in turn, increasing the rate of mortality and morbidity.

Preeclamptic pregnancies are strongly associated with significantly higher medical costs. The maternal costs are related to the extra utility of the healthcare system, more resources used during hospitalization, and likely more surgical spending due to an elevated rate of cesarean deliveries. The infant costs also contribute to a large percentage of the expenses as the babies are prone to preterm deliveries and relevant or causative adverse events. Preeclampsia imposes a considerable financial burden on our societies. It is important for healthcare providers and policy-makers to recognize this phenomenon and allocate enough economic budgets and medical and social resources accordingly.

The true cellular and molecular mechanisms underlying preeclampsia remain largely unexplained, which are assumed to be a two-stage process of impaired uteroplacental perfusion with or without prior defective trophoblast invasion (stage 1), followed by general endothelial dysfunction and vascular inflammation that lead to systemic organ damages (stage 2).

Risk factors for preeclampsia, including race, advanced maternal age, obesity, nulliparity, multi-fetal pregnancy, and co-existing medical disorders, can serve as warnings or markers that call for enhanced surveillance of maternal and fetal well-being. Doppler ultrasonography and biomarkers including the mean arterial pressure (MAP), uterine artery pulsatility index (UtA-PI), and serum pregnancy-associated plasma protein A (PAPP-A) can be used for the prediction of preeclampsia. For women perceived as high-risk individuals for developing preeclampsia, the administration of low-dose aspirin on a daily basis since early pregnancy has proven to be the most effective way to prevent preeclampsia. For preeclamptic females, relevant information, counseling, and suggestions should be provided to facilitate timely intervention or specialty referral. In pregnancies complicated with preeclampsia, closer monitoring and antepartum surveillance including a Doppler ultrasound blood flow study, biophysical profile, non-stress test, and oxytocin challenge test can be arranged. If the results are unfavorable, early intervention and aggressive therapy should be considered. Affected females should have access to higher levels of obstetric units and neonatal institutes. Before, during, and after delivery, monitoring and preparation should be intensified for affected gravidas to avoid serious complications of preeclampsia. In severe cases, delivery of the fetus and placenta is the ultimate solution to treat preeclampsia.

Although the aforementioned theories may provide a reasonable explanation for the results observed in the past studies, the detailed etiology, pathophysiology, and effect of preeclampsia seem complicated, and further research to address the primary etiology and pathophysiology underlying the clinical manifestations and outcomes is warranted.

## Figures and Tables

**Table 1:** Classification of preeclampsia according to gestational age.

Gestational Age	Terminology
GA < 34 weeks	Early-onset preeclampsia
GA ≥ 34weeks	Late-onset preeclampsia
GA < 37w	Preterm preeclampsia
GA ≥ 37w	Term preeclampsia

**Table 2:** Classification of preeclampsia according to severity.

Abnormality	Non-Severe Type	Severe Type
Systolic blood pressure	≥140 mmHg	≥160 mmHg
DBP	≥90 mmHg	≥110 mmHg
Thrombocytopenia (<10^11^/L)	Absent	Present
Abnormal liver function (liver enzymes two times the normal limits)	Absent	Present
Renal insufficiency (serum creatinine level exceeding 1.1 mg/dL or two times the normal limits)	Absent	Present
Proteinuria	Absent or Present	Absent or Present
New-onset headache	Absent	Present
Visual disturbance	Absent	Present
Upper abdominal pain	Absent	Present
Pulmonary edema	Absent	Present
Convulsion/Eclampsia	Absent	Present

**Table 3:** A brief summary of risk factors for preeclampsia.

High-Risk Factors	Moderate/Other Risk Factors
Prior history of preeclampsia Chronic hypertension Pre-existing type I or type II diabetes mellitus Renal disease Autoimmune disease (esp. antiphospholipid syndrome, systemic lupus erythematous) Young maternal age (<25 years)	Nulliparity Advanced maternal age (>35 or >40 y/o) Family history of preeclampsia Long interpregnancy interval (>5 or >10 years) Maternal overweight/obesity (BMI > 30 or 35 kg/m^2^) Multifetal gestation Family history of early-onset cardiovascular disease History of SGA or adverse gestational outcomes Previous miscarriage with same partner Low maternal birthweight of preterm delivery Increased prepregnancy triglycerides Heritable thrombophilia Connective tissue disorder Vaginal bleeding in early pregnancy Gestational trophoblastic disease Drug abuse

**Table 4:** Possible pathways and explanations of mechanisms underlying preeclampsia.

Possible Pathways	Explanations
Failure of maternal vascular adaptation	Some circulating cytokines and growth factors at abnormal levels may inhibit normal calcium signaling events and damage the cell-to-cell contacts of the endothelium. Decreased production or bioavailability of nitric oxide (NO).
Dysregulation of the renin-angiotensin system (RAS)	AT1-AAs may induce calcium signaling and initiate events related to preeclampsia. AT1-AA may be associated with increased TNF-α, indirectly causing damage to the endothelium and end organs.
Oxidative stress	Excessive oxidative stress in the placenta may increase circulating placenta debris, indirectly damaging the maternal endothelial cells. Swollen mitochondria in trophoblasts have been found in preeclamptic models, which is associated with cell apoptosis.

**Table 5:** A comparison between different prediction models of preeclampsia.

Organization	NICE (National Institute for Health and Care Excellence)	ACOG (American College of Obstetricians and Gynecologists)	ISSHP (Iinternational Society for the Study of Hypertension)	FMF (Fetal Medicine Foundation)
Screening method	Based on numbers of risk factors High-risk factors: Previous pregnancy with preeclampsia Chronic hypertension Autoimmune disease Diabetes mellitus Moderate-risk factors: Nulliparity Age ≥ 40 y/o Interpregnancy interval ≥ 10 years Initial BMI ≥ 35 kg/m^2^ Family history of preeclampsia Multifetal pregnancy	Based on numbers of risk factors High-risk factors: Previous pregnancy with preeclampsia Chronic hypertension Autoimmune disease Diabetes mellitus Multifetal pregnancy Renal disease Moderate-risk factors: Nulliparity Age ≥ 35 y/o Interpregnancy interval ≥ 10 years Initial BMI ≥ 30 kg/m^2^ Family history of preeclampsia History of SGA or adverse outcomes Socioeconomic features	Based on numbers of risk factors High-risk factors: Previous pregnancy with preeclampsia Chronic hypertension Autoimmune disease Diabetes mellitus Renal disease Initial BMI ≥ 30 kg/m^2^ Moderate-risk factors: Nulliparity Age ≥ 35 y/o Family history of preeclampsia < 6m sexual relationship before pregnancy Connective tissue disorder	Bayes theorem: to combine the a priori risk from maternal characteristics and results of various biomarkers - MAP (mean arterial pressure) - UtA-PI (uterine artery pulsatility index) - PAPP-A (serum pregnancy-associated plasma protein A)
Detection rate	Preterm: 41% Term: 34%	Preterm: 5% Term: 2%	Not documented	8.2%, 64.0%, 71.8%, and 75.8% at 5%, 10%, 15%, and 20% fixed FPRs
False positive rate	Preterm: 10% Term: 10%	Preterm: 0.2% Term: 0.2%	Not documented	

**Table 6:** Recommendations for administration of low-dose aspirin in women at risk of future preeclampsia.

Organization	ACOG 2018	USPSTF 2021	NICE 2019	ISSHP 2018	FIGO 2019
**Indication**	Recommend in high-risk patients Considered in patients with 1 or more moderate risk factors	Recommend in patients with ≥1 high-risk factors or 2 moderate-risk factors Considered in patients with ≥1 moderate risk factors	Recommend in high-risk patients Considered in patients with 1 or more moderate risk factors	Recommended in high-risk patients	Recommended in high-risk patients or when the risk is ≥1/100
**Timing**	Initiate between GA 12 and 28 weeks (best before GA 16 weeks) and use until delivery	Initiate ≥ GA 12 weeks	Initiate ≥ GA 12 weeks and use until delivery	Initiate ≤ GA 20 weeks (best before GA 16 weeks)	Initiate between GA 11 and 14 weeks and use until GA 36 weeks, delivery or preeclampsia
**Dosage**	81 mg/day	81 mg/day	75–150 mg/day	75–162 mg/day	150 mg/day

**Table 7:** A summary of current management practices of preeclampsia.

Management	Explanation
Blood pressure control	Choices for antihypertensive therapy during pregnancy are limited. The most frequently administered drugs include labetalol, hydralazine, and nifedipine. The former two come in intravenous and oral forms, of which intravenous injections are often used in severe or emergent conditions. Labetalol and nifedipine are more commonly recognized as the first antihypertensive medications for gestational hypertensive disorders.
Seizure prevention	Magnesium is the drug of choice for seizure prevention in preeclamptic and eclamptic cases. It is proven to be superior to other anticonvulsants and is associated with fewer side effects. The mechanism is primarily related to its calcium antagonistic effect and potential to function as an NMDA blocker. It is given with an initial loading dose followed by continuous infusion. Neurologic signs and respiratory patterns should be closely monitored to prevent toxicity.
Delivery and Termination of Pregnancy	The only way to stop or reverse the process of preeclampsia formation is delivery. Therefore, prompt delivery is indicated once the patient reaches term pregnancy. For preterm women with severe disease features, termination of delivery should be strongly considered, but risks higher neonatal morbidities and mortalities due to immaturity. In these cases, cortiocosteroids should be administered for fetal lung maturation before delivery if time allows.
Fluid management	Preeclamptic women often experience fluid overload, which could lead to serious complications such as pulmonary edema. Therefore, unnecessary fluids should be avoided.
Diet management	Most evidence regarding diet management against preeclampsia is not strongly convincing. However, maternal weight control, high fiber intake, probiotics use, calcium and vitamin D supplements, multivitamin and multimineral supplements, and avoidance of a high-salt diet and raw food are considered to be beneficial. A Mediterranean-style diet that is rich in vegetables, fruits, and healthy fats has also been proven to lower the risks of preeclampsia.
Exercise	Aerobic exercise is associated with a reduction of gestational hypertensive disorders as it promotes placentation and a healthier immune reaction in general. The frequency, intensity, type, and time of exercise should be an individualized plan discussed between the patient and physician based on the maternal condition.
Long-term follow-up	Even after delivery and the recovery of preeclampsia, women still bear an increased risk of developing cardiovascular, renal, and hepatic sequelae, along with other chronic diseases. Therefore, long-term follow-up for the patient’s health condition is indicated.

## Data Availability

Not applicable.
